# A model for personalized diagnostics for non-specific low back pain: the role of the myofascial unit

**DOI:** 10.3389/fpain.2023.1237802

**Published:** 2023-10-13

**Authors:** Siddhartha Sikdar, John Srbely, Jay Shah, Yonathan Assefa, Antonio Stecco, Secili DeStefano, Marta Imamura, Lynn H. Gerber

**Affiliations:** ^1^ Center for Adaptive Systems of Brain Body Interactions, George Mason University, Fairfax, VA, United States; ^2^ Department of Bioengineering, George Mason University, Fairfax, VA, United States; ^3^ Department of Human Health and Nutritional Sciences, University of Guelph, Guelph, CA, United States; ^4^ Rehabilitation Medicine Department, National Institutes of Health Clinical Center, Bethesda, MD, United States; ^5^ Department of Rehabilitation Medicine, New York University Grossman School of Medicine, New York City, NY, United States; ^6^ Optimal Motion Inc, Herndon, VA, United States; ^7^ Faculty of Medicine, University of São Paolo, São Paulo, Brazil; ^8^ Department of Medicine, INOVA Health System, Fairfax, VA, United States

**Keywords:** low back pain, myofascial pain syndrome, personalized diagnostics, mechanism-based clinical assessment, biopsychosocial model, myofascial unit

## Abstract

Low back pain (LBP) is the leading cause of disability worldwide. Most LBP is non-specific or idiopathic, which is defined as symptoms of unknown origin without a clear specific cause or pathology. Current guidelines for clinical evaluation are based on ruling out underlying serious medical conditions, but not on addressing underlying potential contributors to pain. Although efforts have been made to identify subgroups within this population based on response to treatment, a comprehensive framework to guide assessment is still lacking. In this paper, we propose a model for a personalized mechanism-based assessment based on the available evidence that seeks to identify the underlying pathologies that may initiate and perpetuate central sensitization associated with chronic non-specific low back pain (nsLBP). We propose that central sensitization can have downstream effects on the “myofascial unit”, defined as an integrated anatomical and functional structure that includes muscle fibers, fascia (including endomysium, perimysium and epimysium) and its associated innervations (free nerve endings, muscle spindles), lymphatics, and blood vessels. The tissue-level abnormalities can be perpetuated through a vicious cycle of neurogenic inflammation, impaired fascial gliding, and interstitial inflammatory stasis that manifest as the clinical findings for nsLBP. We postulate that our proposed model offers biological plausibility for the complex spectrum of clinical findings, including tissue-level abnormalities, biomechanical dysfunction and postural asymmetry, ecological and psychosocial factors, associated with nsLBP. The model suggests a multi-domain evaluation that is personalized, feasible and helps rule out specific causes for back pain guiding clinically relevant management. It may also provide a roadmap for future research to elucidate mechanisms underlying this ubiquitous and complex problem.

## Introduction

Low back pain (LBP) is the leading cause of disability globally with an average prevalence of 39% in the United States (1–3). LBP presents significant financial burden to society (4). Low back and neck pain are the highest contributors to healthcare spending in the US, exceeding $135 billion in 2016 (5). It has been estimated that up to 90% of LBP is non-specific (6, 7), meaning symptoms of unknown origin without a clear, identifiable and specific cause or pathology. This lack of clarity presents a major challenge with diagnosis and appropriate treatment. Consequently, current clinical practice guidelines (8, 9) typically recommend diagnostic triage through physical examination and history taking to rule out underlying “red flags” (e.g., malignancy, fracture or infection), neurological testing to identify radiculopathy, and assessment of psychosocial factors. These guidelines do not offer mechanism-based management strategies targeted to individual patients with LBP who do not present with red flags or radiculopathy, without which poor clinical outcomes are more likely in this population (10). Given the rapidly growing physical and financial burden of LBP, there is an urgent need to identify phenotypes among patients with non-specific LBP (nsLBP) that have the potential for personalized management, and can lead to testable hypotheses about underlying mechanisms.

It is recognized that nsLBP is a complex multifaceted pain condition encompassing biological and psychosocial domains (11). However, the underlying pathophysiologic mechanisms are still poorly understood. A substantial body of literature exists on the association of biomechanical (12–14) and psychosocial factors (15–17) in nsLBP. More recently, the term nociplastic pain has been proposed in the literature (18) to explain the manifestation of neuroplastic maladaptations commonly observed within the nociceptive pathways of nsLBP patients in the absence of verifiable pathology. Significant gaps in our understanding still exist surrounding the complexity of *tissue-level interactions* among the sensory, motor, vascular, lymphatic, immune and sympathetic nervous systems, biomechanical and postural factors, and their relationship to central mechanisms, psychosocial contributors and even environmental factors. Emerging research suggests that all these components merit consideration if we are to be successful in properly diagnosing and treating this multi-factorial problem (19, 20).

In this paper, we describe a new model that seeks to provide a linkage between the tissue-level biological and physiological abnormalities, biomechanical and postural abnormalities, and abnormalities in the central nervous system. Central to this model is the concept of the myofascial unit (19), defined as an integrated anatomical and functional structure that includes muscle fibers, fascia (including endomysium, perimysium and epimysium) and its associated innervations (free nerve endings, muscle spindles), lymphatics, and blood vessels. Our model describes how central mechanisms of segmental sensitization, perpetuated by persistent nociceptive bombardment from somatic or visceral pathologies, can cause downstream dysfunction at the myofascial unit in the low back, and in turn lead to abnormal biomechanics and posture. Segmental sensitization in the spinal cord can be modulated through descending mechanisms, providing a plausible link between psychosocial factors and tissue-level abnormalities.

Our model is informed by clinical observations about nsLBP (including deep, achy and diffuse regional pain, palpable physical findings in affected muscles, altered range of motion, and absence of a nociceptive withdrawal reflex), as well as another non-specific musculoskeletal pain condition: myofascial pain syndrome (MPS) (21–24), whose clinical findings overlap with nsLBP (25). A hallmark of MPS is a localized MTrP, which many clinicians define as a “discrete palpable hyperirritable locus within a taut band of a skeletal muscle that elicits a referral sensation with pressure application” (26). MTrPs are prevalent in the quadratus lumborum muscle of nsLBP patients (25, 27–29). Emerging research is beginning to highlight the key foundational role of central sensitization (CS) and neurogenic inflammation, along with tissue-level interactions in MPS, that include muscle, lymphatics, blood vessels, nerves and fascia, collectively referred to as the “myofascial unit” (19, 30). This conceptual shift away from MTrPs and towards the more comprehensive and integrated idea of the myofascial unit (30) is a step forward in paving the way for a more comprehensive assessment and management strategy informed by multidimensional, patient-specific and mechanism-based diagnostic and treatment criteria. The biological mechanisms at the myofascial unit may offer important mechanistic insight into the clinical manifestation of nsLBP. Our model is also informed by new experimental data that helps link mechanisms of central sensitization, a common component of all chronic pain syndromes, to dysfunction at the myofascial unit through the mechanism of neurogenic inflammation (31–34). Finally, our model is informed by a new understanding of the critical role played by the fascia and loose connective tissue in biomechanical force transmission and postural control (30, 35, 36), and abnormal fascial gliding associated with myofascial unit dysfunction (37).

## Hypothesis: unified model linking the myofascial unit dysfunction to central sensitization and neurogenic inflammation

Several studies have reported soft tissue findings associated with non-musculoskeletal conditions of visceral hypersensitivity such as irritable bowel syndrome and chronic pelvic pain (38–40). This raises the possibility that one contributor to myofascial unit dysfunction may be from primary pathology residing within somatic or visceral tissues (31), such as facet joints, irritable bowel, endometriosis, etc. Although the causal mechanisms are still being investigated, a plausible mechanism is that persistent nociceptive bombardment from these underlying somatic or visceral pathologies can lead to sensitization in the spinal segment, that can subsequently activate dorsal root reflexes that trigger antidromic release of proinflammatory neuropeptides into peripheral tissues to produce neurogenic inflammation and, if allowed to persist (i.e., chronic), can enable complex tissue-level interactions that contribute to the clinical manifestation of myofascial unit dysfunction.

The recently proposed *Neurogenic Hypothesis* (31, 32, 41) postulates the biological foundation of this mechanism. This hypothesis suggests that persistent nociceptive input from a primary pathology of somatic or visceral origin leads to the induction of *central sensitization* within the dorsal horn of segment(s) innervating the pathologic tissue. Central sensitization is a maladaptive state of hyperexcitability within the central nervous system (42, 43). Central sensitization can trigger primary afferent depolarization and dorsal root reflexes within the dorsal horn, leading to the antidromic release of proinflammatory neuropeptides (e.g., substance P, CGRP) peripherally into segmentally-linked tissues (both somatic and/or visceral) via small unmyelinated fibers (31–33). These neuropeptides trigger a cascade of events that include degranulation of mast cells, local vasodilation, plasma extravasation, and the formation of a sensitizing pro-inflammatory biochemical milieu (26). The *Neurogenic Hypothesis* further describes increased efferent activity in both intermediate and ventral horns subsequent to sensitization, mediating enhanced sympathetic and motor efferent activity, respectively. These coexisting pathways create the characteristic proinflammatory biochemical milieu, enhanced sympathetic outflow and motor unit excitability (26).

The N*eurogenic Hypothesis* provides a novel neurobiological framework that explains many of the clinical observations of non-noxious, deep, achy and diffuse pain associated with nsLBP (41). Importantly, it provides the biological plausibility for the commonly observed comorbidity of MPS and nsLBP with pathologies of both visceral and/or somatic origin, in the absence of local injury within the muscle. These mechanisms have been previously reported in a series of studies conducted by Srbely et al. which demonstrate segmentally arranged responses to pressure sensitivity (31) and sympathetic activity (33) in humans using an experimental sensitization model. They have also demonstrated antidromic release of substance P and neurogenic inflammation in segmentally-linked muscles after experimentally-induced osteoarthritis using animal models (32, 34). Support for this notion exists in the literature that has already linked MPS to common injuries and/or somatic pathologies including chronic muscle or ligament strain, intervertebral disc degeneration (44) and/or degenerative joints (45, 46) in addition to chronic non-musculoskeletal pathologies such as metabolic or cancerous conditions (47–49), which are also relevant for nsLBP.

### Downstream effects of persistent central sensitization and neurogenic inflammation

**Figure 1:**
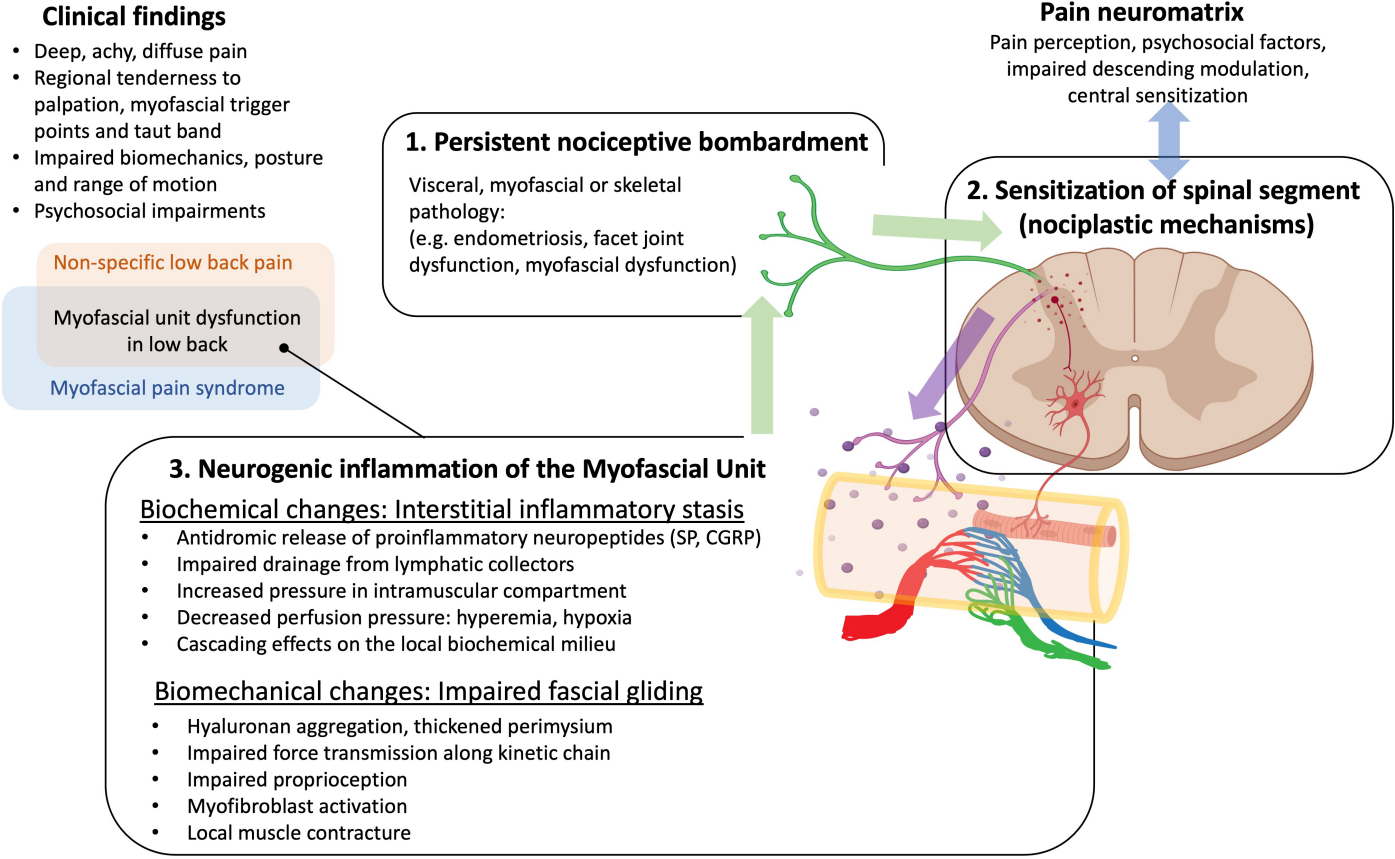
Overarching hypothesis and rationale for our research. Neurogenic inflammation, involving the antidromic release of sensitizing neuropeptides in response to persistent nociceptive bombardment to the dorsal horn provides a link between mechanisms of central sensitization and myofascial unit dysfunction, and can explain many of the clinical findings in myofascial pain and non-specific low back pain. We postulate that neurogenic inflammation as well as other factors can lead to hyaluronan aggregation and/or activation of myofibroblasts that thicken the perimuscular fascia and perimysium and impair fascial gliding, and nociception. Fascial thickening and accumulating cytokines can impair the lymphatic drainage leading to inflammatory stasis that elevates hydrostatic pressure and impairs blood flow, perpetuating the cycle.

A corollary of the Neurogenic Hypothesis suggests that dorsal horn sensitization may influence the activity of intermediate and ventral horns through the release of pro-inflammatory cytokines via activated glial cells (50, 51). This provides the biological rationale for the increased sympathetic and motor activity associated with MPS. Furthermore, persistent neurogenic inflammation within peripheral tissues may lead to pain and dysfunction via *impaired fascial gliding* (30) and *interstitial inflammatory stasis* (52) within the affected myotome (Figure 1). This cascade of events can result in a vicious cycle of pain and inflammation that can persist even after the initial pathology has been resolved, a state that is consistent with the definition of chronic pain.

#### Biomechanical dysfunction and the role of impaired fascial gliding

The fascia is a significant source of pain and dysfunction in MPS. Deep muscular fascia and aponeuroses are richly innervated by small-diameter nociceptive afferent fibers (19, 53, 54), predisposing them to neurogenic inflammatory mechanisms. This feature provides the anatomic rationale for how the fascia may act a potential source of ongoing nociception.

Fascia also plays a significant biomechanical role by providing a continuous anatomical bridge from tissues to organ structures that acts as a scaffold for routing nerves, blood vessels, and lymphatics throughout the body (36, 55). Fascia is integral to force transmission and proprioception. Its rich innervation with free nerve endings and proprioceptors (spindles, Golgi tendon organs) underscores its important role in proprioception (53, 54, 56). It is comprised of both dense and loose connective tissues. The dense portions are mainly formed from collagen types I and III, facilitating the transmission of force from muscle fibers to joints and assisting in the synergistic coordination of complex human movements (57). The loose connective tissue, predominantly consisting of adipose cells, glycosaminoglycans, and hyaluronan, enables the gliding between layers of dense connective tissue, essential for smooth muscle and joint operations. Densification within the fascial compartments has been identified as a significant factor in impaired fascial gliding and dysfunction (35, 58–60). This phenomenon occurs due to the aggregation of glycosaminoglycans, particularly hyaluronan, within the loose connective tissue. As a consequence of this aggregation, the viscosity of the loose connective tissue increases, leading to a reduced range of motion in the myotendinous structures (61). Trauma, overuse, disuse and inflammation (eg., neurogenic inflammation) are primary mechanisms by which the viscosity of hyaluronan can be increased, leading to altered fascial gliding and irritation of unmyelinated nerve fibers (61). This densification was normalized following treatment of spastic muscles in stroke patients with hyaluronidase, an FDA-approved enzyme that breaks down hyaluronan, resulting in increased range of motion (62, 63).

Altered functional gliding of the fascial layers may also lead to irritation of the free nerve endings innervating the fascia, further contributing to pain and discomfort (18). Novel imaging techniques like T1-rho imaging have been used recently in detecting this densification, offering insights into its implications for musculoskeletal health.

#### Biochemical changes and the role of interstitial inflammatory stasis in the clinical manifestation of chronic pain and fascial dysfunction

Neurogenic inflammation can lead to the accumulation of a proinflammatory milieu within the affected tissues to promote pain. Shah et al. (64, 65) were the first to document an abnormal biochemical milieu in active MTrPs using a microdialysis needle technique. The studies revealed that the concentrations of neuropeptides (SP, CGRP, BK), catecholamines (5-HT, NE), and pro-inflammatory cytokines (TNF-α, and IL-1β) were higher in muscle regions demonstrating active MTrPs than latent MTrPs or normal. In addition, pH levels were significantly lower in the active group compared to the latent and normal groups, the latter two of which did not differ.

Accumulating cytokines including TNF-a, IL-1b and IL-6 have been shown to also disable the lymphatic pump as well as stimulating the differentiation of fibroblasts to myofibroblasts to promote fascial contraction. In addition, these cytokines have also been shown to increase the rate of extracellular matrix production and remodeling (fibrotic thickening) of the perimysium over time that lead to impairment of fascial gliding with subsequent stiffness and pain (35, 66). Collectively, these mechanisms if allowed to persist may lead to impaired lymphatic drainage, perimysium thickening and impaired fascial gliding that promotes interstitial stasis of proinflammatory fluids released by neurogenic inflammation (52). This is a potential source of persistent nociception that can facilitate ongoing sensitization and promote chronic pain as exhibited with MPS and nsLBP. Imaging studies using ultrasound (67–69) and MRI (70, 71) have provided additional evidence for increased interstitial fluid near MTrPs.

## Discussion

### Strengths of new model

The primary strength of our proposed model lies in its ability to rationalize clinical observations that highlights important parallels in the clinical manifestation of MPS and nsLBP that suggest common underlying physiologic mechanisms between the two conditions. We propose a novel model for these conditions that draws on the emerging concept of the myofascial unit. This unit is defined as an integrated anatomical and functional structure that includes muscle fibers, fascia and its associated innervation, lymphatics, and blood vessels. The myofascial unit framework offers a more comprehensive perspective of the complex interplay between the different mechanistic pathways that have been linked to the clinical manifestation of MPS and nsLBP. In this way, we offer biological plausibility for the complex overlapping spectrum of clinical findings associated with MPS and nsLBP that have been a persistent source of confusion to clinicians, researchers, and educators and provides rationale for the well-documented comorbidity of MPS and nsLBP with other somatic and/or visceral conditions of pain hypersensitivity that are currently unexplained using existing hypotheses. Our model suggests that structures like facet joints, intervertebral discal structures, and ligaments could be the originators of a vicious cycle of soft tissue pain associated with nsLBP, that can persist even after the underlying pathology is resolved or treated.

The model postulates a vicious cycle between neurogenic inflammation and inflammatory stasis leading to dysfunctional mechanics of the myofascial unit that further exacerbates, and is exacerbated by, biomechanical and postural factors leading to further pain and inflammation. Additional psychosocial factors such as stress, anxiety, depression, sleep and mood disturbances, and pain catastrophizing further contribute to the affective component of chronic pain perception in the pain neuromatrix, and can also modulate descending inhibitory mechanisms that can impact tissue-level abnormalities (Figure 1).

The proposed model enables the integration of central and peripheral mechanisms by drawing on the mechanisms of *central sensitization*, a well-established pathophysiological mechanism observed in all chronic pain syndromes. It prioritizes patient individuality and proposes the use of evaluation tools and imaging methods to investigate the dynamics of fascia gliding, mechanical changes at the myofascial unit level, local vascular changes, local fluid content of tissue, motor unit excitability, and the local biochemical milieu. This approach enhances the ability to identify patient phenotypes, leading to sensitivity and specificity of diagnostic criteria and appropriate mechanism-based treatments that result in improved clinical outcomes.

Finally, our model explains why many different therapeutic modalities, including manual therapies, needling with and without analgesics, acupuncture and complementary and alternative medicine approaches, all have been documented to provide relief yet none of the modalities alone has been shown to have consistent and reproducible evidence of efficacy at Level 1. We postulate that the complex interplay between the different mechanistic pathways between patients may be the primary explanation, further supporting the need for a patient-specific multidimensional assessment protocol that is sensitive to the unique clinical phenotype of each individual subject.

### Transforming current clinical guidelines into proposed multidimensional assessment in the clinic

Current evidence-based clinical practice guidelines for assessment of the patient with LBP (8, 9) emphasize the use of history taking and physical exam to identify “red flags” and rule out serious medical conditions, neurological testing for radicular syndrome, and psychosocial assessment. We advocate that this approach, while a good starting point, is inadequate alone, since it does not provide a systematic approach to assessing other more common forms (i.e., non-specific) of low back pain. Additionally, this approach, diagnosis of exclusion for nsLBP does not provide insight into causal explanations for pain in the absence of obvious tissue pathology. We propose a systematic multidimensional assessment for nsLBP, allowing for more individualized patient assessment driven by underlying mechanisms, assessment of activity limitations, and use of validated patient-reported outcome measures. There have been attempts to classify chronic LBP patients into groups based on clinical characteristics, then further subgrouping into management strategies and interventions (10, 13). However, to date, no single classification system has proven more effective than another for reducing pain and disability in patients with chronic LBP (72). Most of the more successful classification systems integrate individualization and motor control components (13, 14) but are still insufficient.

Our proposed systematic multi-dimensional assessment takes the foundational work to the next level by providing additional insight into the underlying mechanisms, considering patient individuality, and attempting to maintain objective continuity based on a neurobiological framework. To fully capture the amalgamation of neuroscience and biomechanics effecting nsLBP as a system, we are suggesting an expansion of the understanding of pain generators to encompass the idea of an integrated myofascial unit (muscle, fascia, blood vessels, nerve and lymphatics) so as to broaden the clinical evaluation of the MPS patient to include each of these structures.

Based on the mechanistic framework presented above, we propose a structured and comprehensive clinical assessment aligned with the guiding principle that CS is a foundational driving mechanism in the pathophysiology and clinical manifestations of nsLBP. While maladaptive neuroplastic changes in the nervous system, like CS, are evoked by persistent nociception arising from *active* primary pathologies, these maladaptive changes, once established, may persist long after the resolution of the primary pathology.

The primary goals of the comprehensive nsLBP assessment are to (1) assess for the clinical signs of CS and identify any active or resolved primary pathologies that could have contributed to the manifestation of CS; (2) understand biomechanical factors and assessment of activity limitations that can guide management to achieve patient-specific functional goals; and (3) assessment of psychosocial factors (20) that can play a role in descending inhibition, and stressors and protective factors that might indicate maladaptive changes (73).

We recognize that clinical time is limited and often a large number of tests using detailed physical assessment is not practical. Nonetheless, we support the value of learning about the various components that may be assessed to positively identify nsLBP, use it to appropriately plan treatment and possibly identify a need for additional diagnostic testing. The comprehensive assessment includes the following, which may be considered a menu of options to serve as a guide to practicing professions (Table 1):

1. Assessment of pain characteristics:
  a. Systems review to identify underlying active primary pathologies (of visceral and/or somatic origin), serious medical conditions as described in current clinical practice guidelines for nsLBP, and additional pathologies that can contribute to CS, such as osteoarthritis, disc degeneration, and visceral conditions. History and physical examination starts with self-reports of pain, its location and its characteristics. Patient-reported outcomes such as the Oswestry Disability Index and the Roland Morris Questionnaire, Patient-Specific Functional Scale and SF-36 are supported in the literature (74).
  b. Regional assessment that focuses on the identification of chief complaints or patient subjective experience. This involves identification of any involved organs and/or tissues and their neurosegmental connections by assessing dermatomes, myotomes, viscerotomes and sclerotomes for signs of inflammation, injury and/or pathology. While an association between nsLBP and MPS has been shown in the literature, assessment of myofascial tissues is typically not part of the routine evaluation for nsLBP. Many, but not all (75–78), clinicians and investigators agree that an essential characteristic of MPS is the presence of a MTrP, a “discrete palpable hyperirritable locus within a taut band of a skeletal muscle that elicits a referral sensation with pressure application” (78). MTrPs can be active or latent. Active MTrPs are spontaneously painful, and their palpation reproduces the symptomatic pain with a characteristic referral pattern. Latent MTrPs are not spontaneously painful and elicit pain only when palpated or disturbed (26). An extensive review (79) documented that “trigger point, muscle and pain” were evaluations used in 90% of published studies. Pain pressure threshold, range of motion, and manual muscle testing were the most frequent functional objective measures reported. Palpation of muscles in the region with particular attention to the presence/absence of hyper-irritable nodules MTrPs and the presence of taut-muscular bands were common.
  c. Pain phenotyping that aims to determine where on the pain spectrum the patient falls, from acute to chronic, including episodic to persistent, and non-spontaneous to spontaneous (12). A thorough clinical history will enable the clinician to characterize the quality, intensity, duration, frequency, and radiation (if present) of pain. Furthermore, the character of the pain and response to perturbation should be assessed through physical exam. For example, chronic non-specific pain is typically described as a deep achy pain that is poorly localized and diffuse, in contrast to the well-demarcated sharp presentation of acute pain. Furthermore, in contrast to local acute pain, the muscular/myofascial pain associated with chronic nsLBP does not typically induce a nociceptive withdrawal reflex when the tissue is perturbed or challenged via digital pressure during a physical examination.
  d. Quantitative Sensory Testing (QST) techniques to assess for/quantify the presence of windup, a neurophysiologic mechanism commonly associated with CS. Windup shares common mechanisms with CS and is the physiologic manifestation of enhanced temporal summation to repetitive stimuli. A recent systematic review (80) demonstrated significant increases in temporal summation in nsLBP (81, 82). Enhanced temporal summation has also been reported with other chronic musculoskeletal conditions including osteoarthritis, fibromyalgia (83), and MPS. The goal of this stage is to identify affected spinal segment(s) demonstrating enhanced temporal summation, suggesting the presence of either an active nociceptive source or a previously resolved pathology which as evoked maladaptive changes within its neuromeric field. Identifying and resolving an active primary pathology is essential to the long-term resolution of the musculoskeletal component of nsLBP. In addition, treating the spinal segmental sensitization after a resolved primary pathology is also essential for nsLBP. Other QST measures can be used to assess the integrity of the somatosensory system and phenotype pain, including heat and mechanical pain and/or detection thresholds, vibration detection thresholds and Pain Pressure Thresholds (84, 85) using algometry. Use of assessments of CS and autonomic dysfunction as potential estimation of blood flow abnormalities should be considered. This is based on the established segmental somato-sympathetic mechanisms commonly observed in regional pain syndromes.
2. Assessment of body movement:
  a. Movement assessment to determine where on the movement spectrum the patient is (86) and if there is a decrease in optimization of movement, including asymmetry, decreased AROM, hypermobility with or without control, and altered timing of firing. Perform important functional tasks with high interrater reliability or individualized to patient specificity (87) as identified with a patient reported outcome such as the Patient Specific Functional Scale. Active straight leg raise, overhead squat, five time sit to stand, picking up an object from the floor, partial abdominal curl up (strength), side bridge and trunk flexor tests (endurance), Beighton and Brighton (hypermobility), single-legged hop, and lateral step-down, unilateral stance test with eyes open may identify suboptimal tissue loading (14), threat interference, altered movement patterns, loss of balance, asymmetry, altered posture or kinematics, and/or avoidance. Since human movement is highly adaptable to be task specific, patient movement may uncover contributing factors to the clinical manifestations of CS. Follow up with manual muscle testing to confirm or deny observed lack of optimization for weakness and/or neurosegmental involvement.
  b. Range of motion (ROM) assessment. Limitations in range of motion, as well as asymmetry in range of motion and compensatory movements could indicate impaired fascial gliding and densification, or regional pain sensitivity, neurogenic inflammation and/or interstitial inflammatory stasis due to lack of ROM or hypermobility. Most evaluations include range of motion and strength measures of the anatomical segments involved, including range of motion, strength and assessment of triplanar motion of the low back. These are sometimes assessed while performing functional activities of reaching, walking and stooping.
3. Assessment of psychosocial factors:
  a. Current clinical guidelines recommend analysis of psychosocial factors in the evaluation of nonspecific LBP. Psychosocial factors should be evaluated using clinical history and validated PROs to identify potential impact on descending modulation of pain (88). Metrics such as the history of previous episodes (89–91), psychological distress or depression (32–34), fear of pain or movement, and reinjury or low expectations of recovery (92, 93) and a passive coping style (8, 16, 94) and fear avoidance are also often assessed in the comprehensive evaluation of the MPS patient.
  b. In addition to descending modulation, an imbalance of stressors and protective factors (17, 73) may lead to contributory maladaptive changes (allostatic load) that may perpetuate pain perception (95, 96). Evaluation has often included measures of function and quality of life as well as symptoms of fatigue, depression/dysphoria and anxiety. However, the sensitivity of the self-reports and the reliability of the physical examinations were not reported and are inconsistent and do not appear to be based on individualized patient needs.

**Table 1:** Systematic multidimensional assessment of nonspecific low back pain.

**Desired endpoint:** Evaluation for nsLBP should be centered around elements that include assessment of pain characteristics, assessment of movement, and assessment of psychosocial factors. The clinician can select appropriate components and methods from the following menu of options depending upon the appropriateness of the particular patient needs. **Goal 1:** Making sure there is not an identifiable cause of LBP **Goal 2:** Identify the components that lead to a personalized treatment plan or need for additional diagnostic testing.			
(1) Assessment of Pain Characteristics			
	Component	Methods	Interpretation
1.	Underlying primary pathology	Thorough systems review including assessment of underlying (clinical, sub-clinical) visceral and somatic pathology	Identify potential pain generators, including myofascial pain as primary or secondary
2.	Soft tissue assessment	Regional assessment: Musculoskeletal assessment of dermatomes, sclerotomes, myotomes (eg. Pinch/roll)	Identify a neurosegmentally-linked primary pathology (somatic, visceral, sclerotomal)
		Comprehensive physical examination, assessing strength, assessment of symmetries, and palpation for active and latent MTrPs.	Identify neuromusculoskeletal abnormalities, including physical findings of taut band, tender nodules and impaired gliding
3.	Pain phenotype	Comprehensive clinical history of pain onset in patients’ own words and PROs (standard assessments of pain characteristics, mood, affect and sleep) and impact on function in the context of patient's needed and desired activities. (eg Oswestry Disability Index)	Character and quality intensity, duration, frequency (episodic or persistent), and radiation (if present) of pain, indicating regional and/or central sensitization.
4.	Assessment of central sensitization	Assessment of allodynia, hyperalgesia, and quantitative sensory testing (pain pressure threshold, windup ratio) in dermatomes neurologically linked to the primary findings.	Decreased PPT and/or WUR suggests presence of sensitization, enhanced temporal summation/windup
(2) Assessment of Body Movement			
	Component	Methods	Interpretation
5.	Global movement analysis	Assessment of gait, balance, and activities of daily living. Assess symmetry, reaction time, movement completeness, compensation/substitution, efficiencies/ergonomics	Integrated systems view (kinetic chain, sensorimotor control and proprioceptive mismatch). Identify biomechanical factors contributing to movement dysfunction
6.	Joint range of motion	Assessment of range of motion (active, passive, and symmetry), joint stiffness/hypermobility (Beighton/Brighton)	Identify impaired biomechanics and fascia mobility at the affected areas and neurosegmentally.
(3) Assessment of Psychosocial Factors			
	Component	Methods	Interpretation
7.	Descending modulation	Clinical history, medication history and PROs addressing depressive symptoms, anxiety, pain catastrophizing (eg Pain Catastrophizing Scale or Coping strategy questionnaire).	Identify impaired descending inhibition contributing to pain perception, amplification and chronification.
8.	Stressors and protective factors	PROs for lifestyle and environmental factors.	Identify factors associated with allostatic load and cumulative physiological stress, and adaptation or maladaptation

### Implications for future research to improve personalised medicine for back pain

Testing of the conceptual framework to validate the interaction of the three proposed mechanisms, including neurogenic inflammation, impaired fascial gliding and interstitial inflammatory stasis, associated with the clinical manifestation of MPS and nsLBP, will help to establish the mechanistic contributions to these pain syndromes.

Firstly, the proper selection of evaluation tools, assessing their feasibility and ease of use in the clinical settings is critical to developing accurate and reliable databases for the syndrome. Treatment effectiveness can only be assessed if the measurements are acknowledged to be consistent, reliable, sensitive and specific for clinical use. Table 1 proposes a starting point.

The individual elements of our hypothesis at the tissue level are testable in clinical settings using available technologies, and some of these investigations are currently ongoing in our research group. Imaging methods, such as ultrasound and shear wave elastography (37, 67–69), can be used to investigate the dynamics of fascia gliding and mechanical changes at the myofascial unit; photoacoustic imaging and microvascular Doppler can be used to image local vascular changes (97–99); bioimpedance spectroscopy can be a useful technique to investigate local fluid content of tissue (100, 101); high density surface electromyography can be utilized to investigate motor unit excitability (102); and microdialysis can be utilized to investigate the local biochemical milieu (64, 65).

The objectification of pain measures creates opportunities for exploration of multimodal treatments including pharmacological, non-pharmacological, behavioral, and even environmental adaptive approaches. A key limiting factor in the advancement of research in this field is the capacity to quantify changes in central sensitization. While there are current psychophysical approaches to assessing temporal summation, the biosignature, based on the conceptual framework laid out in this paper, should aim to further advance quantitative measures of CS and neurogenic inflammation, levels of Substance P, CGRP, motor unit activity at the endplate and quantitative sensory testing.

Ongoing research studies are developing objective methods to understand the physiology of the myofascial unit. Methods being explored include measures of fascial physical properties including ultrasound imaging and physical examination of movement along with measures of interstitial inflammatory stasis using Doppler imaging of blood flow, and bioimpedance spectroscopy. However, abnormal findings on imaging may not be meaningful for clinical management or prognosis without additional research indicating their utility. Additional research is also needed to better understand the biochemical milieu of the myofascial unit using microanalytic measures of pro-inflammatory cytokines.

The proposed model will have heuristic value if we can show that the evaluation of the different components of the myofascial unit enable us to develop (1) more sensitive and specific diagnostic criteria for nsLBP and (2) appropriate mechanism-based treatments that lead to improvement of clinical outcomes. High quality randomized studies are needed in the future to evaluate if the proposed diagnostic approach leads to improved outcomes compared to the standard of care. If future studies do not support the postulated mechanisms proposed in this model, the model would need to be revised accordingly.

## Data Availability

The original contributions presented in the study are included in the article/Supplementary Material, further inquiries can be directed to the corresponding author.
